# 56 Integrating Cutaneokinematics in Burn Care: A Rehabilitation Best Practice Quality Improvement Project (Phase One)

**DOI:** 10.1093/jbcr/iraf019.056

**Published:** 2025-04-01

**Authors:** Sarah Fischer, James Lenk, Donna Bergkamp, Thomas Resch

**Affiliations:** Ascension Via Christi Regional Burn Center; Ascension Via Christi Hospital - St. Francis; Ascension Health; Ascension Via Christi Regional Burn Center

## Abstract

**Introduction:**

Evidence-based practice recognizes cutaneokinematics as a key concept in development of the rehabilitation therapy plan of care for burn patients. Without sufficient treatment, burn patients are at risk for scar contracture and deconditioning, among other complications. The first phase of this quality improvement project focused on rehabilitation therapy staff knowledge and confidence in applying the concept of cutaneokinematics.

**Methods:**

A before-after project design was used to compare therapist knowledge and confidence prior to and after education and initial use of newly developed cutaneokinematics tools. These tools included a body diagram for accurate depiction of skin involvement and a “burn impact” reference chart detailing key stretches to be performed for each body area affected by burn injury and movements therapists should expect to be impacted. Education was provided using didactic and case study design. An anonymous survey was completed prior to education and again after application of tools in the clinical setting. This survey contained five knowledge questions (multiple choice) and five confidence questions (Likert scale). The results were compared to ascertain whether the education was effective in increasing knowledge, confidence, or both. Significance was assessed using the Chi-squared and Fisher’s exact tests.

**Results:**

Twenty-seven participants completed the pre-survey with only one choosing not to complete the follow up survey. Knowledge in determining stretches for burns that do not cross joints improved (p < 0.001) with therapist confidence in prescribing these stretches nearing significance (p = 0.057). Additionally, therapists were able to identify long term goals for stretching after acute care discharge (p = 0.002) and felt confident in providing patient education (p = 0.034; p = 0.041). However, there was minimal change in therapist confidence in positioning to prevent contracture and providing education on scar management.

**Conclusions:**

Integration of cutaneokinematics tools had a positive effect on rehabilitation therapist knowledge and confidence regarding stretching of smaller, isolated burns and the ability to provide patient specific education to limit complications of burn scar and contracture. A gap was identified in therapist confidence in positioning, as is necessary for splinting or casting. Next steps include development of a simulation lab to reinforce competence of burn stretching and encourage therapist confidence with positioning per cutaneokinematics principles.

**Applicability of Research to Practice:**

Lack of access to high quality, evidence-based therapy interventions place individuals sustaining burn injuries at risk for complications which may severely impair function and quality of life.

**Funding for the Study:**

N/A

